# *“It’s not a time spent issue, it’s a ‘what have you spent your time doing?’ issue…”* A qualitative study of UK patient opinions and expectations for implementation of Point of Care Tests for sexually transmitted infections and antimicrobial resistance

**DOI:** 10.1371/journal.pone.0215380

**Published:** 2019-04-16

**Authors:** Sebastian S. Fuller, Agata Pacho, Claire E. Broad, Achyuta V. Nori, Emma M. Harding-Esch, Syed Tariq Sadiq

**Affiliations:** 1 St George’s University of London, Institute for Infection and Immunity, Applied Diagnostic Research and Evaluation Unit, London, United Kingdom; 2 St George’s University Hospitals NHS Foundation Trust, London, United Kingdom; University of South Florida, UNITED STATES

## Abstract

Sexually transmitted infections (STIs) continue to be a major public health concern in the United Kingdom (UK). Epidemiological models have shown that narrowing the time between STI diagnosis and treatment may reduce the population burden of infection, and rapid, accurate point-of-care tests (POCTs) have potential for increasing correct treatment and mitigating the spread of antimicrobial resistance (AMR). We developed the *Precise* social science programme to incorporate clinician and patient opinions on potential designs and implementation of new POCTs for multiple STIs and AMR detection. We conducted qualitative research, consisting of informal interviews with clinicians and semi-structured in-depth interviews with patients, in six sexual health clinics in the UK. Interviews with clinicians focused on how the new POCTs would likely be implemented into clinical care; these new clinical pathways were then posed to patients in in-depth interviews. Patient interviews showed acceptability of POCTs, however, willingness to wait in clinic for test results depended on the context of patients’ sexual healthcare seeking. Patients reporting frequent healthcare visits often based their expectations and opinions of services and POCTs on previous visits. Patients’ suggestions for implementation of POCTs included provision of information on service changes and targeting tests to patients concerned they are infected. Our data suggests that patients may accept new POCT pathways if they are given information on these changes prior to attending services and to consider implementing POCTs among patients who are anxious about their infection status and/or who are experiencing symptoms.

## Introduction

Sexually transmitted infections (STIs) continue to be a major public health concern in the UK, with approximately 420,000 diagnoses made in England alone in 2017 [1]. Young people continue to bear the burden of STIs in the UK; diagnoses among 15–24 year-olds are twice as high in men and seven times as high in women compared to other age groups [1]. Diagnoses of *Chlamydia trachomatis* (CT) and *Neisseria gonorrhoeae* (NG) infections continue to be the most common STI diagnoses among men-who-have-sex-with-men (MSM) [1]. Other curable STIs, such as *Mycoplasma genitalium* (MG), have not traditionally been routinely tested for in National Health Service (NHS) sexual health clinics (SHCs) as there is limited provision of accurate, molecular based testing for MG, despite evidence of this infection causing onward health consequences such as endometritis, cervicitis, pre-term birth and pelvic inflammatory disease in women [2,3].

The *Precise* study is a collaboration between clinicians, epidemiologists, microbiologists, social scientists and industry experts to develop and evaluate rapid nucleic acid amplification tests (NAATs) -based Point of Care tests (POCTs) for multiple STIs and AMR detection. A social science programme, including qualitative research and patient and public involvement, was developed within the *Precise* study to incorporate patient and clinician opinions on the potential designs and implementation of new rapid NAAT-based POCTs for CT, NG, MG and Trichomonas vaginalis (TV) diagnosis and AMR detection in MG and NG infections.

Laboratory-based NAATs are the current gold standard for CT and NG testing [4–6]. Laboratory NAATs usually allow clinical staff to provide patients with results in about a week [7]; some non-laboratory-based NAATs in development promise sensitivity and specificity comparable to laboratory NAATs and provision of results in 30 minutes or less [8], allowing for accurate diagnosis and treatment at the point of care. Epidemiological models have shown that narrowing the time between diagnosis of infection and treatment may reduce the burden of infection in the population [9], and rapid, accurate NAAT-based POCTs have the added potential for increasing likelihood of correct treatment and mitigating the spread of antimicrobial resistance (AMR) in STIs [10]. NG and MG infections in particular have shown high potential for resistance, and reducing the spread of resistant infections is a priority globally and in the UK [10–13].

Implementation of rapid POCTs within SHCs is likely to necessitate changes to clinical pathways [14], such as patients providing samples for POCTs prior to a clinical consultation [15], and in so doing, change patients’ experiences and potentially their perspectives of SHC attendance. While we have some understanding of clinician and Industry perspectives for priorities for the development of POCTs for STIs [16–19], there is less knowledge of patient perspectives on how implementation of these technologies may change patient care [20], and no published research on patient perspectives for implementing AMR POCTs in SHCs. Here we report on our qualitative study conducted with patients in SHCs across the UK, as part of the *Precise* social science programme.

## Methods

We planned qualitative research consisting of informal interviews with clinicians and semi-structured in-depth interviews with patients in six SHCs in the UK. Two data collection stages were planned with a year of analysis between them; we hypothesised that this would allow for iterative adaption of our topic guides to include new areas of enquiry following our mid-term analysis.

UK SHCs were selected for inclusion in the study based on geographic location, access to a wide range of patients, and willingness to participate. Geographic locations were chosen to represent different areas of the UK, in order to better understand if location was associated with differing provision or expectations of care. Different types of services were also chosen, including clinics that have been associated with innovative care and/or testing models alongside clinics with more traditional models of service delivery.

Prior to patient interviews, consultant-level sexual health clinicians at each of the six clinics were interviewed to assess: their views of the need for STI and AMR detection NAAT-based POCTs for their patient population, the ideal design(s), and how they might implement the tests. Consultant clinicians were chosen as ideal to interview as they are the most senior doctors in the service and, as such, are involved in making key decisions on how their clinical services are implemented. Clinician interviews included collaborative development of flowcharts of potential clinical pathways that would enable test implementation at each service. Verbal descriptions of these pathway changes were incorporated into our interview topic guides to allow for realistic portrayal of clinical pathway changes patients could then address in their interviews. Interview topic guides are included as Supporting information (S1, S2 and S3 Files).

In the patients interviews, participants were told by the interviewer that the design of the tests, specifically which infections were bundled onto each test cartridge, and if the NAAT-POCT included AMR as well as infection detection, would influence how much time they might spend in clinic to wait for results, and if needed, treatment. It was explained that ‘reflex testing’, or testing for alternate causes of infections in those patient that were found to be negative in their first test would result in waiting for an additional 30 minutes or longer at clinic (e.g. those negative for CT/NG might then receive a test for TV and/or MG). Reflex testing might also be necessary if AMR was not included within an infection detection test, which would mean that patients found positive for NG or MG infection would need to wait an extra 30 minutes for the result of an AMR POCT test to guide their treatment.

In UK sexual health clinics, patients’ reported sexual behaviours, including the gender(s) of their recent sexual partners, are used as a proxy for determining the complexity of their clinical needs. Thus sexual behaviour data are used to triage patients into care pathways, which include the type of healthcare professional they will see (e.g. nurse or doctor) and the potential types of tests they will receive. Based on this, we chose to sample patients in each clinic based on their age and the gender(s) of their reported recent sexual partners, rather than sexual identity. Our reporting throughout reflects this choice.

All participants invited into the *Precise* social science study were patients of participating NHS SHCs who reported symptoms of bacterial STI infection and so were at high risk for infection. This gave us a greater chance of interviewing patients that had an STI; many participants received their STI test results before the time of their interview. This allowed us to conduct in-depth semi-structured interviews with men and women with a confirmed recent STI(s) as well as those who have tested negative; knowing patients’ infection status allowed for exploration of specific circumstances of how their care might have been altered if they had access to the POCTs under development.

Although research has shown that there are important ethnic differences in knowledge and experience with sexual health care [21], it was not possible for us to achieve ethnic diversity in the sample that would allow analysis of those differences. The majority of the population in the UK is White (59.8% in London; 88.8% in Yorkshire; 95.4% in South West England and 96% in Scotland) [22,23]; our sample is reflective of this.

Table 1 presents our purposive sample frame. We expected N≥54 patients across the six sites (n≥9 per clinic).

**Table 1:** Purposive sample frame, per clinic.

	Men reporting exclusively heterosexual behavior	Men reporting sex with men	Women reporting sex with men	Total
**Ages 16–24**	1	1	1	3
**Ages 25–35**	1	1	1	3
**Ages 35–44**	1	1	1	3
Total	3	3	3	9

Patients were initially screened by a healthcare professional for eligibility and then approached by a clinic-based researcher at the time of their clinic attendance with an invitation to participate in an interview. Healthcare professionals conducting recruitment for the study described the study to patients as University research for the benefit of improving UK NHS service provision in SHCs. Potentially eligible patients were invited to provide their first name, ethnicity, contact details (telephone and/or email address), and their preference for time/day of contact by the research team. These patients were given a patient information sheet and at least 24 hours to consider their participation before being contacted by the interviewer to arrange an interview appointment in the SHC where they had been recruited, or over the phone. Patient interviews were scheduled at a time of the patients’ preference, including out of regular working hours and on weekends. At the time of the interview appointment and prior to the interview, patients gave their full informed consent to participate. Participants each received a £20 voucher for their participation.

SF, a man in his early 40s with over 15 years’ experience in qualitative research, conducted the first set of interviews, and AP, a woman in her early 30s, with over 5 years’ experience in qualitative research, conducted the second set of interviews. Both SF and AP presented themselves to healthcare professionals and potential participants in participating clinics as University researchers that were independent from the company producing the NAAT-based POCTs discussed in the interviews. Both interviewers encouraged positive, negative and indifferent views of the tests to be expressed.

Interviews were audio recorded and transcribed verbatim. Transcripts were then checked for accuracy (cleaned) against the audio recording by the interviewers (SF, AP). A content analysis approach was used to capture and uncover substantive meanings within the dataset. Data were analysed using a thematic approach. SF and AP looked for common themes across the dataset. Transcripts were coded thematically in NVivo 11. Framework was used as a tool to organise themes, as this approach allows for reading themes across and within cases, giving opportunity for both in-depth case study analysis and explanatory analyses based on comparison of themes across the dataset [24]. SF led the analysis and selected initial themes, AP then reviewed the entire dataset and generated and assigned themes independently to transcripts to improve reliability. Finally, all themes were agreed on by both SF and AP prior to finalising the analysis and drafting the manuscript.

We report the research in accordance with the consolidated criteria for reporting qualitative research (COREQ) checklist (Supporting information: S4 File).

### Ethics

Ethical approval was given in June 2015 by London Bridge Research Ethics Committee, reference 15/LO/0535: *Developing patient-centred*, *rapid*, *Point-of-Care testing including antimicrobial resistance markers for specialist sexual health services in the NHS*: *the Precise study social science programme*.

## Results

Six clinics across UK participated: London (n = 3), Devon, Yorkshire, and Eastern Scotland. London SHCs included two central London locations, including one associated with implementation of novel testing pathways and modern designs to encourage patient attendance. A total of 148 patients agreed to be contacted by the research team for an interview, of which 63 patients (42.6%) completed an interview. Identification of potentially eligible patients was conducted in different ways by members of the healthcare team in each of the six clinics (e.g., some patients were invited before eligibility screening and then asked eligibility questions, whereas others did the opposite), and so we cannot provide meaningful data on patients who declined to provide contact information to the study team.

Data were collected in two stages: June 2015 –February 2016 and February 2017-August 2017. Two interviews were unusable due to recording errors. Of 61 useable patient interviews, 18 women reporting sex with men, 17 men who reported exclusively heterosexual behaviour and 26 men reporting sex with men, participated.

The mean age for heterosexual male participants was 25 (range 17–37), for men reporting sex with men was 30 (range 19–40), and for female participants was 28 years (range 20–41). No patients aged 16 years or between 42–45 years of age participated. The majority of participants (50/61) were White, almost one-third (27.9%; 17/61) of participants were students (n = 9 London, n = 4 Yorkshire, n = 2 Devon, n = 2 Scotland), and four were unemployed and not in education. Full demographics details for all participants are shown in Table 2.

**Table 2:** Demographics details for all participants*.

Gender	Female (%)	Male (%)	Total	Column %
	18 (29.5%)	43 (70.5%)	61	100%
**Age**				
16–24	6 (31.6%)	13 (68.4%)	19	31.1%
25–34	9 (31%)	20 (68.9%)	29	47.5%
35–44	3 (23%)	10 (76.9%)	13	21.3%
**Ethnicity**			61	100%
Asian or Asian British	3 (75%)	1 (25%)	4	6.5%
Black/Black British	1 (33.3%)	2 (66.6%)	3	4.9%
Mixed race	0 (0%)	2 (100%)	2	3.3%
Other (not specified)	1 (100%)	0 (0%)	1	1.6%
White/White British	13 (26%)	37 (74%)	50	82%
Prefer not to answer	0 (0%)	1 (100%)	1	1.6%
**Sexual behaviour**			61	100%
Men reporting sex with men	-	26 (100%)	26	42.6%
Men reporting exclusively heterosexual behaviour	-	17 (100%)	17	27.9%
Women reporting sex with men	18 (100%)	-	18	29.5%
**Occupation**			61	100%
Employed	10 (25%)	30 (75%)	40	65.6%
Student	6 (35.3%)	11 (64.7%)	17	27.9%
Unemployed	2 (50%)	2 (50%)	4	6.5%

* All data are self-reported

During interviews, we asked participants to describe their most recent clinic visit. Following participants’ description of their recent clinic experience, we discussed the NAAT-based POCTs. We initially gave very little information about the tests, asking their opinions of the potential to receive their test results within their same visit to clinic generally, and then explored participants’ willingness to wait for NAAT-based POCT results in a scenario where the new pathway would extend their time spent waiting in the clinic. We next discussed participants’ opinions on the potential new NAAT-based POCT pathways. We report these data here in the order they appeared in the interview. The following main themes emerged, and are explained in detail using quotes from the respondents, below: initial impressions, willingness to wait for POCT results, reflex testing and recommendations for implementation. These descriptive data led to our explanatory analysis, exploring how patients’ levels of experience influenced their opinions and expectations for their sexual healthcare. Finally, we report participants’ recommendations for implementation of the tests.

**Fig 1:**
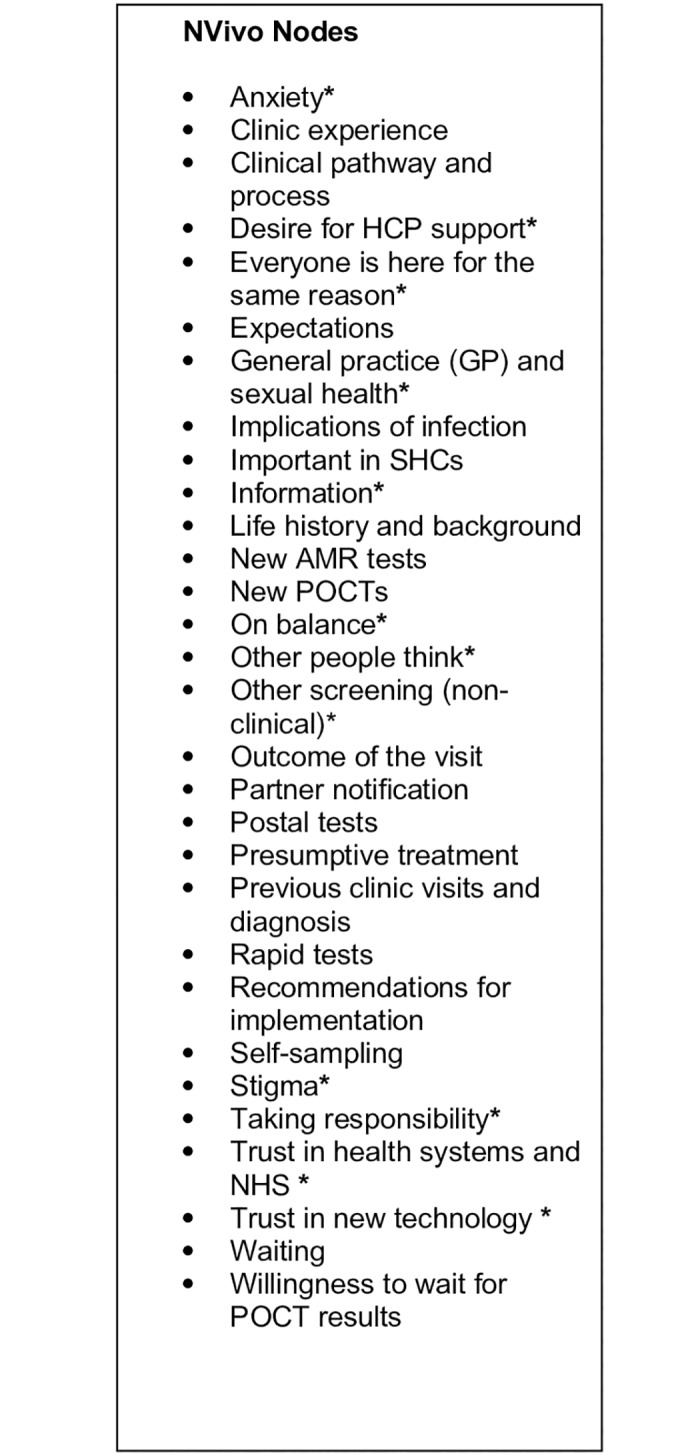
NVivo nodes. * Respondent driven themes.

The full list of themes is shown in Fig 1. Asterisked nodes represent themes that emerged independently of the questions asked by the interviewer. Non-asterisked codes represent themes that follow directly from the topic guides (Supporting information: S2 and S3 Files).

### Initial impressions

Most participants were enthusiastic about the potential to receive diagnosis and treatment within one clinic visit; many initially assumed that the POCTs would mean that their time in clinic would be less than current practice.

“Well I’d be happy with that, because I mean you’d be in and out quickly, and everyone wants to know that day. I—I wanted to know that day.”

-Man reporting exclusively heterosexual behaviour age 18, London (LA02)

“I think it’s very good, yeah, both for patients and doctors and nurses. I think it’s quicker, easier, to get results back—yeah, no, a very good idea.”

– Man reporting exclusively heterosexual behaviour age 32, Yorkshire (B08)

#### Turn-around-time and willingness to wait for results

Following discussion of initial impressions of the tests, participants were given a brief explanation on the NAAT-based POCTs: the test will diagnose CT, NG, TV, and MG infections and the turn-around-time from sample into the cartridge to a diagnostic result will be ≤30 minutes. Participants were then told that their time in clinic was likely to be longer than currently, to accommodate the test turn-around-time and results delivery. Most participants described this scenario as being preferable to current practice.

“So I’d rather just sit an hour and a half one day and not have to waste the time travelling [back] to the clinic … if I can just travel one time and be seen in one to two hours rather than over two days [that] is, I think, better.”

-Woman reporting sex with men age 22, London (LA01)

“I think for any test you feel apprehensive and you feel uncomfortable. So any shortening of that time from test to solution is a positive thing in my eyes.”

– Man reporting exclusively heterosexual behaviour age 22, Yorkshire (B01)

We then went on to describe the scenario of reflex testing, where patients might be asked to undergo a second test following the results of the first NAAT-based POCT. Participants were mixed in their response to wait an additional 30 minutes for reflex testing, with many questioning why all infections and AMR were not able to be included on a single cartridge. Several participants discussed that reflex testing would be considered acceptable in specific circumstances, but not in others.

When analysing the data for factors that explain differences in participants’ opinions of willingness to wait for the results of the proposed NAAT-based POCTs, a strong theme emerged about the context of sexual healthcare seeking. One young man explained that, for him, waiting in clinic for a 30-minute test, or a series of 30-minute tests (e.g. in the case of reflex testing) would be time spent doing something “important” and was thus preferable to “sitting and waiting” for laboratory results at home:

“[The POCTs are] much better than sitting and waiting around, because no one enjoys that. And, it’s not a ‘time spent’ issue, it’s a ‘what have you spent your time doing’ issue, and if a lot of that time is filled up with something that you or I feel is important, then that’s fine with me.

- Man reporting exclusively heterosexual behaviour age 22, Yorkshire (B01)

When investigating what “something you or I feel is important” may mean, we found that many participants explicitly described situations where they would be willing to wait if they perceived themselves at risk for infection. Participants described feelings of being at risk for infection if they had genital symptoms or engaged in sexual risk-taking (e.g. condomless sex), such as this young man:

“I mean, if I was in a position where I’d had unprotected sex with people and felt like I was at the risk of STIs, then, yes, I’d be more than happy to wait … I think it all comes from gauging how much at risk I am at the time as to how much amount of time that I’m willing to put into getting tested and getting it sorted.”

-Man reporting sex with men age 20, Devon (P09)

Although the NAAT-POCT pathways we described were an acceptable change from current practice for most participants, some felt that spending additional time waiting for results at the clinic would not be acceptable, such as this young woman recruited at one of the London clinics:

“Probably [I would prefer] less time [in clinic] and a text message in a few days. … Yeah. Well if I’m honest, I’ve not really had much of a problem with the waiting time of getting the results [by text message]…”

- Woman reporting sex with men age 20, London (LA03)

Most participants that preferred the current testing and results system at the clinic to the proposed new NAAT-based POCT pathways expressed that waiting at clinic for 30 minutes or more from sampling to results was too time consuming. Some, such as the young woman quoted above, felt that waiting for about a week for a text message with her results was acceptable; she did not see the need to change the current care pathway. Another participant felt that the turn-around-time was unacceptable compared with the instant HIV test he had taken several years earlier:

“No, that’s long, my friend. That’s very, very, very long. Yes. If it is possible to get it within an average of 15 minutes, now we’re fine.”

- Man reporting exclusively heterosexual behaviour age 33, Yorkshire (B02).

Some clinicians in participating SHCs directed which infections patients tested for, based on their self-reported sexual history, while others took a more patient-led approach, allowing patients to request a “full screen” of potential STIs and HIV. Regardless of the type of clinic attended, most participants felt that testing for some infections with the POCTs was either preferable or an acceptable compromise to waiting for all their STI testing results to return from the laboratory in about a week:

“I mean, it’s a shame it couldn’t all be done at the same time so you wouldn’t have to wait, but if that was the way that it’s done, then I would rather have some results back straight away and just have to wait for the other ones…”

–Men reporting sex with men age 27, London (LA09)

However, there were some that felt it was not acceptable to receive their diagnoses at different times. These patients felt that the process was “half a job” and “incomplete” until they received all their results and so would prefer the current system:

“… I’d just think what’s the point in getting some of it in half an hour when you can’t have it all in half an hour … I don’t like doing things half—half a job.”

- Woman reporting sex with men age 21, Yorkshire (B07).

“It’s—that’s sort of an incomplete process, you’d want to know the full score, as you’re there. I think it’s just like as I said initially, you don’t want to wait for so long to hear about some other things that were sort of still pending. You’d want to know everything straight away, and that’s why—because that’s why you went to the clinic.”

– Man reporting exclusively heterosexual behaviour age 30, London (LA11)

#### ‘Experienced’ and ‘less-experienced’ patient views

Among our participants, we identified a subgroup of ‘experienced patients’. Although all of the participants in this study were patients of sexual health services, we defined ‘experienced patients’ as a discrete group of participants who described one or more of the following: screening for infections often, living with a chronic condition, acquiring a high level of familiarity with NHS settings through accessing family planning, child health services, or similar, and/or having a medical background or working in the NHS. The majority of our participants were classed as ‘experienced’; 21.3% (13/61) of participants had little or no prior experience with sexual health clinics and/or health systems more generally and so were described as ‘less experienced patients’ (see Table 3 for demographic details of ‘experienced’ and ‘less experienced patients’). Men reporting exclusively heterosexual behaviour were the largest group among the ‘less experienced patients’ (61.5%).

**Table 3:** Demographic details: ‘Experienced’ and ‘less experienced’ patients.

	Experienced patients (%)	Less experienced patients (%)	Total
**All participants**	48 (%)	13 (%)	61
Age*			
16–24	14 (77.8%)	4 (22.2%)	18
25–34	21 (72.4%)	8 (27.6%)	29
35–44	12 (92.3%)	1 (0.7%)	13
Gender*			
Female	15 (83.3%)	3 (16.7%)	18
Male	33 (76.7%)	10 (23.3%)	43
Transgender/transsexual	0 (0%)	0 (0%)	0
Sexual behaviour*			
Men reporting exclusively heterosexual behaviour	9 (52.9%)	8 (47.1%)	17
Women reporting sex with men	15 (83.3%)	3 (16.7%)	18
Men reporting sex with men	24 (92.3%)	2 (7.7%)	26
**Recruitment sites**			
London	30 (85.7%)	5 (14.3%)	35
Yorkshire	6 (60%)	4 (40%)	10
Devon	7 (87.5%)	1 (12.5%)	8
Scotland	5 (62.5%)	3 (37.5%)	8

* Self-reported data

‘Experienced patients’ often built expectations of sexual health services and opinions of the POCTs based on experiences with previous medical visits; one female participant described her expectations based on experiences with medical appointments for herself and her children:

“I would have been happy to wait another half an hour [after the initial test]. Because when I kind of think about other things that I’ve had done, like screening for breast lumps, you accept that, I would accept that there, I’d been there for two hours before, waiting for the whole, the biopsy, the mammogram, the ultrasound and the various talking to the consultants before and afterwards. … Equally, my children, I’d taken them to the paediatrics’ centre and I’ve waited for two hours for them, but I’d gone away with a clearer picture of what’s going on. So I’d be happy with that…”

-Woman reporting sex with men age 41, Devon (P07)

‘Experienced patients’ were also more likely to be sceptical that a 30-minute turn-around-time would mean that their results would be delivered within that timeframe:

“But then would that half-an-hour be half-an-hour if there’s if there’s a load of people waiting? …will everyone get half-an-hour treatment?”

-Woman reporting sex with men age 31, (LA06)

Based on their previous experiences of using SHCs, these participants envisioned issues that may potentially arise once the NAAT-based POCT was in clinical use:

“It seems good I guess. My first reaction was, well, if the clinic has one [machine], and you see people every five minutes, you’re going to end up with a massive queue just to wait half-an-hour for each test.”

-Woman reporting sex with men age 28, (B09)

Within this sub-group, acceptance of the NAAT-based POCTs and their opinions of receiving diagnoses at different times often were based on their knowledge of sexual health:

“I think for me personally, I’d be okay because I know the people that I have sex with, like our group statistically, chlamydia and gonorrhoea are the most common.”

-Woman reporting sex with men age 21, (LC12)

### Recommendations for implementation

When looking at recommendations for implementation of POCTs, there was significant diversity among our participants with regard to the knowledge and/or experience of pathways and processes present in SHCs. Our data show that experience and knowledge of SHCs plays a role in how patients form their opinions and expectations of these services. These opinions and expectations led to participant recommendations for the implementation of POCTs. Participants frequently expressed their desire for information about steps involved in point of care testing, estimations of duration of clinic visits being available prior to attending and the rationale behind AMR testing.

“I think if I knew from the start how long I was going to be there, I’d be like, ‘okay that’s fine.’ … But if I’d have got to what I thought was going to be the end of my appointment … and then they were like, ‘you’ll have to wait half an hour.’ I’d say, ‘why couldn’t you have told me this before I was like thinking I was going to leave in five minutes from now, and I’m sat here for an extra 30 minutes?’”

- Woman reporting sex with men age 28, Yorkshire (B09)

“… I think as long as you understand the process and why this is happening, then, yes, I would have no issue with that whatsoever. … You know, it’s your health so it’s putting that time aside to make sure that you get the right results and the right treatment is really important. But yes, understanding the process, I think, is the key part of that.”

- Man reporting sex with men age 35, Devon (P08)

Participants’ suggestions to increase acceptability of new pathways for POCTs also included targeting tests to those that are concerned they are infected (e.g. those presenting as symptomatic).

“…maybe it’s kind of tailored to people who already have a suspicion of having gonorrhoea or chlamydia… if you go in with suspicions or to find out about gonorrhoea, chlamydia, then it’d be more helpful.”

–Man reporting sex with men age 26, London (LA08)

Furthermore, a number of patients said they would like to have a choice to either wait for the results in the clinic, or return later that day for their results:

“I imagine, you’re okay to just go and get a coffee somewhere, either in the hospital or outside, and come back in 30 minutes? I don’t know if they need you to stay in the building necessarily, but I think just by giving people options just being sort of kind and creating a nice atmosphere and be like, like you got 30 minutes until your result, kind of, do what you please. So yes, for me, I would prefer it if I didn’t have to stay in the waiting room.”

–Man reporting sex with men age 27, London (LB07)

## Discussion

### Context within the existing literature

This is the first study of patients’ opinions and priorities for POCTs for STI and AMR detection implementation in UK SHCs. Previous studies have focused on patients’ views of home-sampling and/or home-testing [25,26]; however, these studies have shown that patients have different opinions and concerns around these services than they do of SHCs. Several studies have shown a general acceptability of waiting times for same day results, even if that means extending that time from 20 minutes to up to two hours [27–29]. None of these studies have been conducted in the UK and none have reported the context of patients’ decisions to wait for their results and so our ability to understand how to implement NAAT-based POCTs for STIs is limited.

Miners *et al*. explored preferences for STI testing services and found that their participants (patients of SHCs) preferred to receive their results on the same day, placed the highest importance in choice of where to attend for STI testing on receiving specialist knowledge and testing for all infections at the same time [30]. Several of our participants similarly indicated preference for SHCs based on their specialist care, and discussed testing for as many infections as possible on the same clinic visit. Interestingly, most of our participants were unconcerned about receiving results for different infections at different times, despite their overall lack of familiarity with POCTs (including the HIV rapid test). Our data strongly indicate that receiving results at different times was less than ideal, but still preferable to current practice.

Although it might be expected that patients generally prefer their test results faster than is currently possible, our data show that patients’ views are highly contextual, with a strong them among our participants that NAAT-based POCTs and willingness to wait for results is contingent on the reason(s) for their clinic attendance. In the treatment of curable STIs specifically, NAAT-based POCTs reduce the duration of infection through prompt treatment and expedited partner notification, reduce overtreatment and may mitigate the development of AMR through targeted prescribing [18]. However, the potential for NAAT-based POCTs to increase testing coverage has been questioned, given the existing cultural and health barriers to accessing sexual health services [18]. Our study shows that it is possible to critically engage with patients about their opinions of NAAT-based POCTs for STIs, and their priorities and pathways for implementation of these new technologies, which may increase acceptability and thus use of these services in the future.

Debates in medicine around the volume of information that is made available to patients lack consensus [31]. While patients are believed to almost always wish to receive as much information as possible [31,32], research has shown that care providers are rarely aware of this inclination [31,33–36]. Many studies have indicated that patients’ dissatisfaction with the provision of information from healthcare providers affects the quality and outcomes of care, including patients’ satisfaction with health services [31,32,37–44]. Patients’ satisfaction is, in turn, associated with their compliance with treatment directives [35,45–46], which is itself an important indicator of patient outcomes.

### Limitations

We did not reach our purposive sample goal for men reporting exclusively heterosexual behaviour between 16–24 and 35–44 years of age, and women reporting sex with men between 35–44 years of age, therefore these age groups are under-represented in this sample. Men reporting sex with men are over-represented in the sample, following our attempts to increase enrolment following underrepresentation in the first sub-study; response by men reporting sex with men was higher than anticipated in the second sub-study. As a result of our limited data from men reporting exclusively heterosexual behaviour between 16–24 and 35–44 years of age, and women reporting sex with men between 35–44 years of age, we may have more limited theoretical generalisability than intended. As men reporting exclusively heterosexual behaviour were the largest group among the ‘less experienced’ patients, it would be advantageous for the study to have a better insight into their views. We did however achieve theme saturation in our sample, indicating that new participants would have been unlikely to express different opinions.

Our agreement of 42.6% (63/148) of patients that provided contact information and went on to complete an interview is low as compared with other qualitative interview studies, which may have biased our sample. We complied with recommendations for best ethical practice for conducting research in healthcare settings by having healthcare professionals at each participating clinic identify potentially eligible participants as this allows patients to refuse approach for anything other than their medical care. However, this means that we are unable to provide meaningful data on patients who declined to provide contact information to the study team. Healthcare professionals in each of the six clinics identified patients in different ways; some asked every patient in the waiting room if they would like to participate (regardless of potential eligibility) and others asked only potentially eligible patients to participate. However, we can see that there was a high level of diversity in attrition across clinics in the time period between acceptance of the contact form and participating in an interview: 31% (5/16) of Yorkshire-based patients, 54.5% (42/77; 60%, 52%, 45% in each clinic) of all London-based patients, 70% (20/29) of Devon-based patients and 69.2% (18/26) of Scotland-based patients who provided contact information did not go on to complete an interview. The majority of patients that did not complete an interview after our attempts to contact them passively refused (97%, 114/117). Failure to respond to our calls/emails to schedule an interview or failure to answer their phone or show up in person for a scheduled interview were considered passive refusal.

Interviewers may influence data collection in human subjects research; we considered this in our analysis by initially conducting separate analyses for each set of interviews. When we examined our dataset to explore the potential differences between the two sets of interviews we could not find any significant differences. Additionally, although the interviewers presented themselves as neutral towards the technology, it is possible that some participants saw the interviewers as representatives of the NAAT-based POCTs, which may have caused some to mitigate their stated opinions of the tests during the interviews.

## Conclusions

Our study shows patients’ willingness to wait in clinic was often dependent on their self-assessed risk for infection. Insight into patients’ priorities for care, and their suggestions that specific, directed messaging may allow acceptability of various changes related to adoption of NAAT-based POCTs in SHCs gives concrete guidance for implementation of these tests. In particular, patients recommended targeting tests to those that are concerned they are infected and giving patients a choice to either wait for the results in the clinic, or return later that day for their results. Adhering to these suggestions for NAAT-based POCT implementation may lead to good acceptability of new clinical pathways for these diagnostics. We recommend further research when these tests are made available, to test these theories in practice.

## Supporting information

S1 File Clinician interview topic guide. (DOCX)

S2 File Interview topic guide 2015. (DOCX)

S3 File Interview topic guide 2016. (DOCX)

S4 File COREQ checklist. (DOCX)
