# Supplementary material for: “It’s not a time spent issue, it’s a ‘what have you spent your time doing?’ issue…” A qualitative study of UK patient opinions and expectations for implementation of Point of Care Tests for sexually transmitted infections and antimicrobial resistance
Source: PLoS One. 2019 Apr 16;14(4):e0215380. doi: 10.1371/journal.pone.0215380 (PMC6467401; doi:10.1371/journal.pone.0215380)
Supplement: S1 File — (DOCX) [file pone.0215380.s001.docx]

Clinician interview topic guide (KPIs)

1. Describe the existing clinical pathway for **symptomatic and asymptomatic** patients at *this clinic*

- Confirm all steps and timing of each:
  - - Registration
    - Consultation
    - Clinical exam
    - Sample collection & preparation
    - Health promotion counselling
    - Microscopy
    - Off site (lab) sample processing
    - Results management (data entry and notification)
    - Results counselling (pos only)
    - PN
- Anything else?

1. Discussion of how the Atlas io™ POCT would affect pathways
   - **Which configuration of the following pathogens would be included in your ideal STI POCT?**

Insure all cartridge options available are discussed, e.g.:

- - Ng/Ct AMR
  - Ng/Ct/TV/Mg
  - AMR standalone
  - Ng/Mg AMR
  - TV/Ct
- Probe:
  - Talk to me about the reasons behind this choice
  - Would inclusion of AMR testing make a difference in your choice of pathogens to include?

1. **Describe the clinical pathway changes following on this POCT adoption**
2. **What impact do you think this test would have on your patients?** Are there specific patient groups that you think would benefit more than others? Less than others?
   - Probe:
     - What are the specific benefits of the POCT you describe? Drawbacks?
