# Supplementary material for: “It’s not a time spent issue, it’s a ‘what have you spent your time doing?’ issue…” A qualitative study of UK patient opinions and expectations for implementation of Point of Care Tests for sexually transmitted infections and antimicrobial resistance
Source: PLoS One. 2019 Apr 16;14(4):e0215380. doi: 10.1371/journal.pone.0215380 (PMC6467401; doi:10.1371/journal.pone.0215380)
Supplement: S2 File — (DOCX) [file pone.0215380.s002.docx]

**Interview topic guide 2015**

**Title:** Developing patient-centred, rapid, Point-of-Care testing including antimicrobial resistance markers for specialist sexual health services in the NHS: the Precise study social science programme

**Name of Researcher:** Dr Sebastian S Fuller, Chief Investigator: Precise social science programme, St. George’s University of London, Cranmer Terrace London SW17 0RE

Research question: What understanding and experiences do patients have of sexual health clinic(s) in England? Are patients’ understanding and experiences correlated to their risk factors or location of the service(s)?

Research question: How do patients feel that their clinical experience may change with the introduction of POC testing including AMR markers?

Introduction: We are asking patients to help us design this test in the best way possible. One of the ways we are doing this is to have a better understanding of your experiences of sexual health clinics and what is important in your visit to clinic. Before we talk about that, I am hoping to get to know a little bit about you first.

1. Life history / framing
   1. Please tell me a bit about yourself.

- What is the most important thing you can tell me about yourself?
  - Probes:
    - Hobbies/interests
    - Goals
- Tell me about your family.
  - Who do you include in your family? Partner/spouse? Father/mother? Grandparents? Children?
- What sorts of things do you do with your day? / Describe a typical day in your life.
  - Probes:
    - Are you in school/university?
    - What do you do for money?

1. Clinical experience:
   1. Please describe your visit to the clinic on the day you were asked to take part in this study.

- Probes:
- How did it go/ how did you feel about the visit?
- How long were you waiting to be seen? Was this expected /unexpected?
- Which infections were you tested for?
- What was the primary reason you came here today?
  1. Tell me about another time you went to the sexual health/STI clinic.
     - Probes:
       - Which sexual health clinics have you been to?
         1. Do you prefer one over the others? What are some of the reasons for this (if yes).
       - How often do you visit the sexual health clinic?
       - What are some of the reasons you have had for attending the clinic?
  2. How were your previous experiences at the sexual health clinic(s) different than today’s visit? How were they the same?
     - Probes:
       - Wait time?
       - Consultation: how long? What was it like? Did you see a doctor?
       - Was one experience better/worse than the other? Is there a clinic you prefer? What do you think has led you to feel this way?
  3. Describe the ways that your sexual health clinic experience(s) could be improved.
     - Probes:
       - What do you think is an essential outcome from your visit to the clinic? / What are you expecting from your visit?
       - Is there anything that you experienced at clinic that you think are not necessary?

1. Factors in acceptability of POCT with AMR

Now let’s talk about ‘point of care testing’ for STIs. When something is called a point of care test, this means that it is a test you would have to go to the clinic for (just as you have done previously) but you would get your test results within that clinic visit.

- 1. General thoughts:
     - What do you think about the possibility of getting your results in the same visit?
     - Are there any circumstances that you would not want to get your test results while you are in clinic?
  2. Acceptability of POCT for some infections but not others
     - The rapid tests that are being developed are for four infections: Gonorrhoea, Chlamydia, Mycoplasma, and Trichomonas. If the doctor or nurse feels that you are at risk for any other infection (i.e. syphilis, herpes) you will still have to wait for the results of those tests from the lab. What do you think about the possibility of getting some of your test results at the same time as your clinic visit but waiting a week or so for other test results? How important is it that you receive your results all at the same time?
  3. Trust in POCT results
     - Do you think you would feel the results from a test that gives you results in the same clinical visit will be as good as those that get sent to the lab? Why/why not?
  4. Acceptability of waiting for AMR results/ receiving AMR results at the POC
     - If you were found to have a gonorrhoea or mycoplasma infection it would be possible to test for antibiotic resistance of those infections. This means that the doctor would know what the best antibiotic is to treat that infection.

In which circumstances would you be willing to wait an additional 30 minutes for the results of this test?

- 1. Acceptability of waiting for infection diagnosis at the POC
     - If you were able to get your test results today, but it meant you had to spend more time in clinic, how much longer would you wait for your results? Half an hour? An hour? Longer?
     - Would there be some times that you were more willing to wait, or would wait longer than others? Which circumstances would make a difference, if any?
  2. Acceptability of potential changes to clinical pathway
     - One of the ways that clinics may choose to use the tests we are developing is to ask patients to provide either a urine or swab sample for testing before they see a doctor. This would mean you would fill in the normal form when you get to clinic, someone would review what you wrote and then you would be given either a swab or a urine pot. How do you feel about that?
       - Probe: self-sampling; giving sample before seeing DR

1. Anything else?
2. Thanks
   1. Reimbursement
