# Supplementary material for: “It’s not a time spent issue, it’s a ‘what have you spent your time doing?’ issue…” A qualitative study of UK patient opinions and expectations for implementation of Point of Care Tests for sexually transmitted infections and antimicrobial resistance
Source: PLoS One. 2019 Apr 16;14(4):e0215380. doi: 10.1371/journal.pone.0215380 (PMC6467401; doi:10.1371/journal.pone.0215380)
Supplement: S3 File — (DOCX) [file pone.0215380.s003.docx]

**Interview topic guide 2016**

**Title:** Developing patient-centred, rapid, Point-of-Care testing including antimicrobial resistance markers for specialist sexual health services in the NHS: the Precise study social science programme

**Name of Researcher:** Dr Sebastian S Fuller, Chief Investigator: Precise social science programme, St. George’s University of London, Cranmer Terrace London SW17 0RE

**Research questions:** What are patients’ views and preferences for the design of point-of-care tests for bacterial STIs and antimicrobial resistance in NHS specialist health services?

What understanding and experiences do patients have of NHS sexual health clinic(s)? Are patients’ understanding and experiences correlated to their risk factors or location of the service(s) they access?

How do patients feel that their clinical experience may change with the introduction of POC testing including AMR markers?

**Introduction:** We are asking patients to help us design this test in the best way possible. One of the ways we are doing this is to have a better understanding of your experiences of sexual health clinics and what is important in your visit to clinic. Before we talk about that, I am hoping to get to know a little bit about you first.

1. Life history / framing
   1. Please tell me a bit about yourself.

- Do you have a (partner/boyfriend/girlfriend)? If yes, how long have you been together?
  1. Describe a typical day in your life.
     - Probes:
       - Are you in school/university?
       - Do you have children/dependents?
       - What do you do for money (e.g. work)?
       - Would you consider yourself to be ‘busy’? Why/why not?

1. Clinical experiences:
   1. Please describe your visit to the clinic on the day you were asked to take part in this study.

- Probes:
- What was the primary reason you came here today?
- How did it go/ how did you feel about the visit?
- Which infections were you tested for?
- How long were you waiting to be seen? Was this expected /unexpected?
  1. Tell me about the reasons you have had for attending sexual health clinics in the past.
     - **How often** do you visit sexual health clinics?
       - Some people **test for STIs regularly**. Is this something you do?
  2. Tell me about another time you went to the sexual health/STI clinic.
     - Which (other) sexual health clinics have you been to, if any?
     - (If yes) where were they? (**geographic location**)
     - **How long ago** was this visit?
     - What were the **reasons for your visit**?
  3. How were your previous experiences at sexual health clinic(s) different than your experience at (recruiting clinic)?
     - Tell me about your **reason for attending** the clinic at that time.
     - Were there **structural** differences (e.g. waiting room configuration, availability of toilets) between (recruiting clinic) and (previous clinic)? If so, did these differences have any effect on your experience?
     - Were there differences in your experience with **staff members** between (recruiting clinic) and (previous clinic)? If so, did these differences have any effect on your experience?
     - Is there **anything else** that you can remember being different between these clinics?

1. Alternate modes of STI testing: POCTs and postal tests

- Now let’s talk about ‘point of care testing’ for STIs. When something is called a point of care test, this means that it is a test you would have to go to the clinic for (just as you have done previously) but you would get your test results within that clinic visit.

**Have you had a test like this before (e.g. rapid or POCT for HIV)?**

1. If yes:

- Describe your experience: why you went to the clinic, how you were feeling about your reason for attending (e.g. were you anxious at all?)
- What did you think about your experience? (Expected/unexpected? Good/bad? Why/why not?)
- How did you feel about receiving your test result at the time of your visit?
- Do you think you would feel the results from a test that gives you results in the same clinical visit will be as good as those that get sent to the lab? Why/why not?

1. If no rapid/POCT experience:
   - - Have you heard of rapid tests, or point of care tests before this?
     - What do you think about the possibility of getting your results in the same visit?
     - Are there any circumstances that you would not want to get your test results while you are in clinic?
     - Do you think you would feel the results from a test that gives you results in the same clinical visit will be as good as those that get sent to the lab? Why/why not?
2. Some clinics provide patients with test kits that you can order online, have sent to your house. You would then receive directions on how to take your own (urine or swab/cotton bud) sample and send it back to the clinic for testing. **Have you ever used online or postal STI test kits before?** If yes:

- Describe your experience: why you ordered the test, how you were feeling about your reason for testing (e.g. were you anxious at all?)
- What did you think about your experience? (Expected/unexpected? Good/bad? Why/why not?)
- How did you feel about testing this way? Were there any positive/negative things about this way of testing as compared to going to clinic? Which did you prefer and why?
- Do you think you would feel the results from a test that you sent away for will be as good as those that get sent from the clinic? Why/why not?

**If no** online or postal test experience:

- Have you heard of postal STI tests before this?
- Would you consider getting a STI test sent to you in the post? Why/why not?
- Do you think you would feel the results from a test that you sent away for will be as good as those that get sent from the clinic? Why/why not?

1. Acceptability of POCT for some infections but not others
   - - The rapid tests that are being developed are for three infections: Gonorrhoea, Chlamydia, and Mycoplasma. If the doctor or nurse feels that you are at risk for any other infection (i.e. syphilis, herpes) you will still have to wait for the results of those tests from the lab. **What do you think about the possibility of getting some of your test results at the same time as your clinic visit but waiting a week or so for other test results?** How important is it that you receive your results all at the same time?
2. Willingness to wait for infection diagnosis and treatment at clinic
   - - If you were able to get your test results today, but it meant you had to spend more time in clinic, how much longer would you wait for your results? Half an hour? An hour? Longer?
       - Would there be some times that you were more willing to wait, or would wait longer than others? Which circumstances would make a difference, if any?
     - If you needed to wait for your results but it meant that if you were found to be infected you would be able to leave having taken your treatment, would you be willing to spend more time at clinic?
       - Would there be some times that you were more willing to wait, or would wait longer than others? Which circumstances would make a difference, if any?
3. Anything else?
4. Thanks
   1. Reimbursement
